# Conservative versus accelerated rehabilitation after rotator cuff repair: a systematic review and meta-analysis

**DOI:** 10.1186/s12891-021-04397-0

**Published:** 2021-07-24

**Authors:** Umile Giuseppe Longo, Laura Risi Ambrogioni, Alessandra Berton, Vincenzo Candela, Filippo Migliorini, Arianna Carnevale, Emiliano Schena, Ara Nazarian, Joseph DeAngelis, Vincenzo Denaro

**Affiliations:** 1 Department of Orthopaedic and Trauma Surgery, Campus Bio-Medico University, Via Alvaro del Portillo, 200, Rome, Trigoria 00128 Italy; 2 Research Unit of Measurements and Biomedical Instrumentation, Campus Bio-Medico University, Via Alvaro del Portillo, 200, Rome, Trigoria 00128 Italy; 3 Center for Advanced Orthopaedic Studies, Carl J. Shapiro Department of Orthopaedic Surgery, Beth Israel Deaconess Medical Center, Harvard Medical School, Boston, MA USA

**Keywords:** Conservative, Accelerated, Rehabilitation, Protocol, Rotator cuff, Rotator cuff repair

## Abstract

**Background:**

The purpose of this systematic review and meta-analysis is to compare the conservative and accelerated rehabilitation protocols in patients who underwent arthroscopic rotator cuff repair in terms of clinical outcomes and range of motions at 3, 6, 12, and 24-month follow-up.

**Methods:**

According to PRISMA guidelines, a systematic review of the literature was performed. For each included article, the following data has been extracted: authors, year, study design, level of evidence, demographic characteristics, follow-up, clinical outcomes, range of motions, and retear events. A meta-analysis was performed to compare accelerated versus conservative rehabilitation protocols after arthroscopic rotator cuff repair. The retear rate, postoperative Constant-Murley score and range of motions at 3, 6, 12, and 24 months of follow-up were the outcomes measured.

**Results:**

The search strategy yielded 16 level I-II clinical studies. A total of 1424 patients, with 732 patients and 692 in the accelerated and conservative group, were included. The average age (mean ± standard deviation) was 56.1 ± 8.7 and 56.6 ± 9 in the accelerated and conservative group. The mean follow-up was 12.5 months, ranging from 2 to 24 months. The meta-analysis showed no statistically significant differences in terms of retear rate between the groups (*P* = 0.29). The superiority of the accelerated group was demonstrated in terms of external rotation (*P* < 0.05) at 3-month follow-up; in terms of forward elevation, external rotation, abduction (*P* < 0.05), but not in terms of Constant-Murley score at 6-month follow-up; in terms of forward elevation (*P* < 0.05) at 12-month follow-up. No significant differences between the two group were highlighted at 24-month follow-up.

**Conclusions:**

No statistically significant differences in the retear rate among the accelerated and conservative group have been demonstrated. On the other hand, statistically and clinically significant differences were found in terms of external rotation at 3 and 6 months of follow-up in favour of the accelerated group. However, no differences between the two groups were detected at 24 months follow-up.

## Background

Rotator cuff (RC) tears represent a significant reason for orthopaedic examination due to their negative impact on the working class’s quality of life [1–4]. The broad interest in RC disease is because pathogenesis is still unclear [5–14], the management remains challenging [15–18], and the patient lifestyle can be scanty [19–23].

Management of RC tears depends on the tear’s characteristics, such as tear severity, location and extent of the injury, tendon retraction, and muscle condition. Treatment of RC can be conservative or surgical [24–28]. Surgery is performed after trauma or a conservative treatment failure to improve shoulder function and relieve pain [29–31]. Among surgical techniques, arthroscopy is the most commonly performed because of minor trauma to the deltoid due to smaller incisions, less postoperative pain, and the possibility of addressing concomitant disorders [32–45]. As a result of the increasing incidence of RC ruptures and the risk of failure after surgical repair, questions have been raised regarding the cost-benefit ratio of surgical treatment compared with conservative management [2, 3, 46–53]. The immobilisation with the sling, the physiotherapist’s role, and the best postoperative week for reintroducing shoulder movement have been widely investigated [24, 29, 54–73]. The best rehabilitation protocol aims to restore shoulder function, allowing the tendon healing process to prevent retear events [60, 74]. To achieve this goal, both conservative and accelerated rehabilitation protocols have been proposed. The first protocol requires a restrained arm during the first six weeks. The rationale for conservative rehabilitation protocol is based on evidence from animal studies showing that the tendon healing process takes 4 to 16 weeks [75, 76]. It is common practice to apply a sling immobilisation in abduction wrap to reduce potential stress at the suture level and to improve the blood flow quantity at the scar [42, 77–83]. However, there are concerns about stiffness after prolonged immobilisation [84–86]. Recent studies have demonstrated that prolonged immobilisation may result in biochemical tissue changes, such as variation in water and glycosaminoglycans concentration, the regularity of collagen cross-bridges, fatty infiltration, and fibres orientation [65, 71]. Therefore, in stiffness-prone patients, an accelerated protocol that gradually reintegrates movement before the sixth week is advocated to achieve better clinical results [87]. However, accelerated rehabilitation protocol seems to increase the risk of tendon retear that occur from 20 to 90 % of cases [64, 80, 88–91].

Even though many systematic reviews are available, the evidence is limited for the low statistical sample and the high heterogeneity among the included studies [4, 53, 92–98]. Since further clinical trials with a longer follow-up have been published recently, this review aims to compare the rehabilitation protocol and the accelerated protocol at different time-points. Therefore, the present study aims to compare the conservative and accelerated rehabilitation protocols in patients who underwent arthroscopic RC repair in terms of clinical outcomes and range of motions (ROMs) at 3, 6, 12, and 24-month follow-up.

## Materials and methods

### Search strategy and study selection

According to PRISMA 2020 guidelines, a systematic review was performed to evaluate accelerated or conservative rehabilitation’s potential benefits in patients undergoing arthroscopic RC repair [99]. Conservative rehabilitation has been defined as a complete immobilization, whereas accelerated rehabilitation gradually reintroduces movements before the sixth week. The analysis has been executed since the beginning of the Cochrane Central Register of Controlled Trials (CENTRAL), MEDLINE, EMBASE, and Google scholar databases until March 31, 2020. The combination of free-text terms and Medical Subject Headings (MeSH) in the title and abstract was used to perform the research. The search strategy was built on the application of Boolean logic operators to the following keywords: (“rotator cuff” OR “rotator cuff tear” OR “rotator cuff repair”) AND (rehabilitation OR “postoperative rehabilitation” OR exercises OR “physical therapy” OR “physical therapies” OR “rehabilitation protocol” OR “rehabilitation program” OR “accelerated rehabilitation” OR “early rehabilitation” OR “conservative rehabilitation” OR “delayed rehabilitation” OR “slow rehabilitation”). Three independent reviewers (U.G.L., L.R.A. and V.D.) had verified the suitability of each article published in a peer-reviewed journal for the relevance of title and abstract to the objective of this study without excluding any journal. Records also include the listed references from the original record as another possible source of relevant trial reports. To increase the study’s strength, only level I-II studies based on the Oxford Centre of EBM were selected and involved in the present research [100]. Studies without abstract or meaningful information were excluded during the study selection process. The independent reviewers conducted an accurate full-text reading of the chosen articles, obtaining data to reduce selection bias.

To be included in this review, eligible studies had to meet the following inclusion criteria:

(i) A comparison between conservative and accelerated rehabilitation protocol;
(ii) A primary arthroscopic RC repair must be performed in patients of both study groups;
(iii) Reported at least one of retear rates, clinical scores, and range of motions;
(iv) Level I-II articles published in a peer-reviewed magazine or presented at a conference.

Exclusion criteria were:

(i) A comparison between one of the two forms of rehabilitation and healthy control;
(ii) Other RC repairs (i.e. tendon reconstruction, arthroplasty, tendon transfer or revision);
(iii) Lack of enough data for extraction;
(iv) Reviews, case reports, articles on animals, cadaver, or in vitro researches, biomechanical reports, technical notes, letters, and instructional studies.

### Data extraction process

For each article included in the study, the following data has been extracted: authors, year, study design, level of evidence, sample size both at baseline and at final follow-up, losses at follow-up, number of patients in the accelerated and conservative group, sex, age, follow-up, clinical outcomes (Constant-Murley score (CMS), Simple Shoulder Test (SST) score, and American Shoulder and elbow surgeons (ASES) score), ROMs (forward flexion, internal rotation, external rotation, internal rotation in abduction, external rotation in abduction, abduction), visual analogue scale (VAS) score, retear events, and exercises performed during the rehabilitation period for both groups.

### Quality assessment

Two independent reviewers (L.R.A and U.G.L.) assessed the risk of bias for each included study. Review Manager (RevMan, version 5 for Windows; Cochrane Information Management System) was used as risk of bias assessment tool. Following methods recommended by The Cochrane Collaboration, a domain-based evaluation (random sequence generation; allocation concealment; blinding of participants, personnel and outcome assessors; incomplete outcome data; selective outcome data reporting and other sources of bias) was performed [101]. The following judgments were used: low risk, high risk, or unclear (either lack of information or uncertainty over the potential for bias). Kappa statistics were used to assess inter-rater reliability between data extraction and quality assessment [102].

The GRADE (Grading of Recommendations Assessment, Development and Evaluation) guidelines were used to assess the critical appraisal status and quality of evidence of the included randomised controlled trials. The combination of four factors (i.e., study design, study quality, consistency, and directness) provided whether the evidence’s quality was high, moderate, low, or very low. We downgraded the evidence quality from’ high quality’ by one level for serious risk of bias, inconsistency, indirectness of evidence, imprecision of effect estimates or potential publication bias [103].

### Meta-analysis

A meta-analysis was performed of all included studies to compare accelerated versus conservative rehabilitation protocols after arthroscopic RC repair. The retear rate, postoperative CMS and ROMs at 6, 12, and 24 months of follow-up were the outcomes measured. Review Manager (RevMan, version 5 for Windows; Cochrane Information Management System) was used to determine the treatment effect’s magnitude. An I^2^ index evaluated the heterogeneity of the principal analysis. I^2^ index describes the percentage of the whole diversity between studies that are made by heterogeneity. According to the Cochrane Handbook for Systematic Reviews of Interventions, the interpretation of the I^2^ for heterogeneity was as follows:

- 0–40 %, was not important.
- 30–60 %, represented moderate heterogeneity.
- 50–90 %, represented substantial heterogeneity.
-  75–100 %, represented considerable heterogeneity.

A fixed-effect model in the data synthesis was adopted when heterogeneity values were ≤ 60 %; otherwise, a random-effects model was used.

Continuous variables were reported as mean ± standard deviation, rounded to a decimal plane. In all studies, *P* < 0.05 was considered statistically significant. For each statistically significant outcome in terms of CMS and ROMs, the achievement of the minimal clinically important difference (MCID) between the two groups was evaluated to determine clinical reliability.

## Results

### Search results

**Fig. 1:**
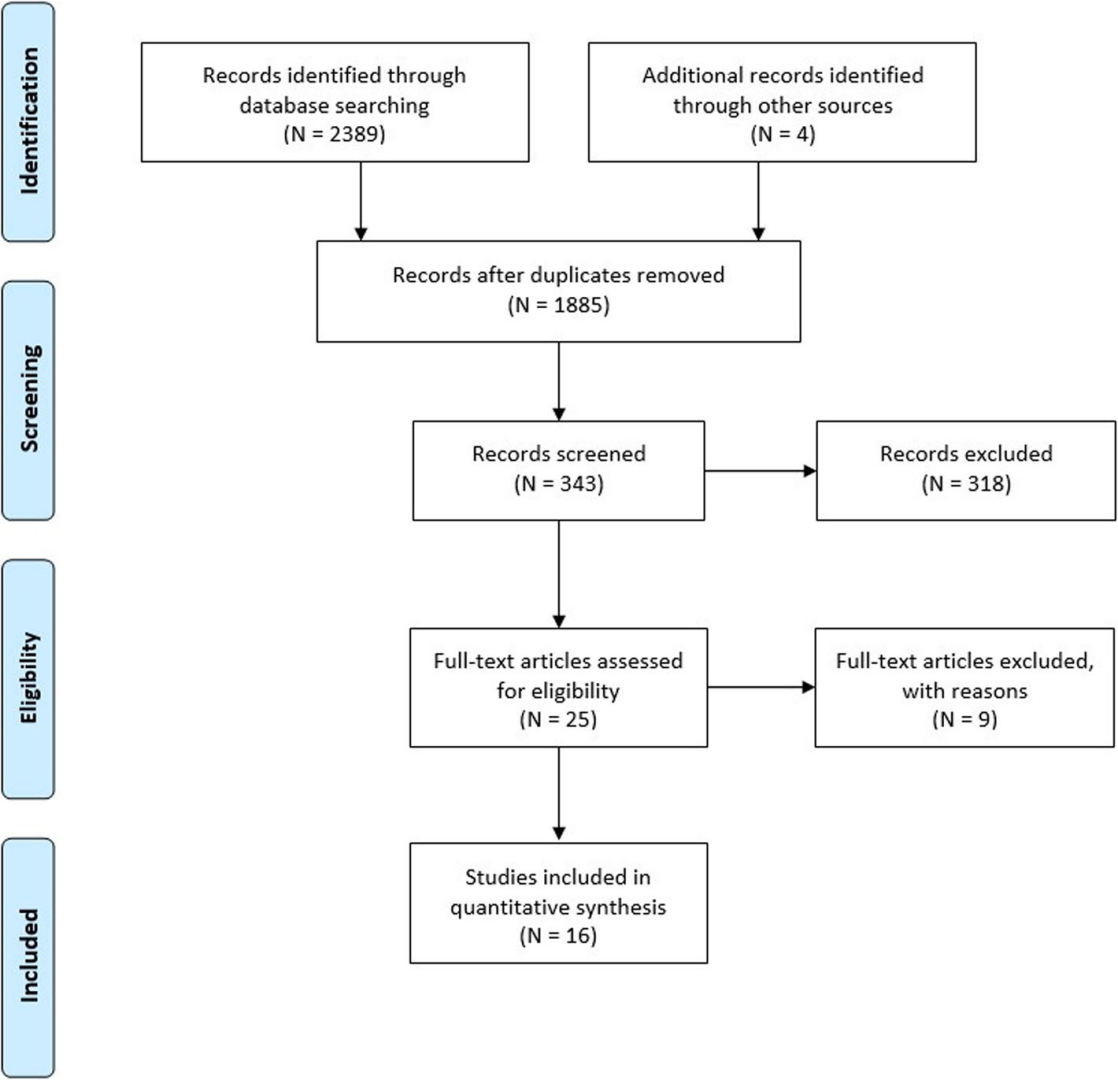
Preferred Reporting Items for Systematic Reviews and Meta-Analyses (PRISMA) diagram representing individual study inclusion after applying the study algorithm

The research strategy has comprehensively yielded 2393 results. After removing duplicates, 1885 articles were filtered by the study design (i.e. level I-II clinical studies). Among the selected 25 clinical studies, four articles were excluded because they compared passive ROM exercises and continuous passive movement [104–107]. Another four clinical studies were excluded because they compared a supervised and self-assisted rehabilitation protocol[55–57, 108]. Conservative rehabilitation was defined as complete immobilization, whereas accelerated rehabilitation was a gradual reintroduction of movement before week 6. Therefore, one study was excluded because it did not fulfill these definitions [109]. Hence, at the end of the thorough investigation by the reviewers, 16 of the 25 studies met the inclusion criteria [29, 61, 63, 64, 67–73, 77–80, 110] (Fig. 1).

### Data extraction

#### Demographics

A total of 1424 patients (776 males and 648 females) were considered, with 732 patients in the accelerated group and 692 in the conservative group. The average age (mean ± standard deviation) was 56.1 ± 8.7 and 56.6 ± 9 in the accelerated and conservative group. The mean follow-up was 12.5 months ranging from 2 [77] to 24 [63, 72, 78, 79] months. Study characteristics are reported in Table 1.

**Table 1:** Demographics

Authors (Year, level of evidence)	No of final patients (No of initial patients – losses at follow-up) ^a^	No of patient in the accelerated group (AG) and conservative group (CG)	Sex	Average age (mean ± standard deviation)	Follow-up (months)
Arndt et al. [61] (2012, II)	92 (100–8)	AG (49) CG (43)	M 34; F 58	54.6 ± 9.8	16
Cuff et al. [70] (2012, I)	68 (68 − 0)	AG (33) CG (35)	AG (M 18; F 15) CG (M 20; F 15)	AG: 54.8 ± 15.9 CG: 56.3 ± 15.6	12
De Roo et al. [110] (2015, I)	130 (130–0)	AG (79) CG (51)	AG (M 48; F 31) CG (M 41; F 10)	AG: 64.6 ± 10.0 CG: 65.1 ± 9.7	4
Düzgün et al. [71] (2011, II)	29 (29 − 0)	AG (13) CG (16)	M 3; F 26	AG: 55.9 ± 7.8 CG: 56.6 ± 11	4
Düzgün et al. [29] (2014, II)	40 (42 − 2)	AG (19) CG (21)	AG (M 2; F 17) CG (M 4; F 17)	AG: 57.7 ± 7.8 CG: 57.2 ± 10.1	6
Jenssen et al. [73] (2018, I)	118 (120–2)	AG (60) CG (58)	AG (M 37; F 23) CG (M 32; F 26)	AG: 53.8 ± 10.1 CG: 54.3 ± 11.3	12
Keener et al. [63] (2014, I)	114 (129 − 15)	AG (61) CG (53)	-	AG: 54.8 ± 6.3 CG: 55.8 ± 6.3	24
Kim et al. [64] (2012, I)	105 (117 − 12)	AG (56) CG (49)	AG (M 26; F 30) CG (M 18; F 31)	AG: 60.1 ± 9 CG: 60 ± 10.4	12
Klintberg et al. [72] (2009, II)	14 (22 − 8)	AG (7) CG (7)	M 9; F 5	53.5 ± 6.9	24
Koh. et al. [77] (2014, I)	88 (100–12)	AG (40) CG (48)	M 44; F 44	59.9	2
Lee et al. [67] (2012, II)	64 (85 − 21)	AG (30) CG (34)	AG (M 21; F 9) CG (M 20; F 14)	AG: 53.5 ± 7.8 CG: 53.85 ± 7.2	12
Mazzocca et al. [68] (2017, II)	58 (73 − 15)	AG (31) CG (27)	AG (M 21; F 10) CG (M 19; F 8)	AG: 55 ± 8 CG: 54 ± 7	12
Raschhofer et al. [69] (2017, II)	29 (30–1)	AG (14) CG (15)	AG (M 10; F 4) CG (M 9; F 6)	AG: 54.4 ± 3.0 CG: 58.4 ± 3.5	6
Sheps et al. [78] (2015, II)	165 (189–24)	AG (97) CG (92)	AG (M 57; F 40) CG (M 58; F 34)	AG: 55.4 CG: 54.9	24
Sheps et al. [79] (2019, I)	206 (206 -0)	AG (103) CG (103)	AG (M 65; F 38) CG (M 66; F 37)	AG: 55.5 CG: 56.2	24
Tirefort et al. [80] (2019, I)	80 (80 − 0)	AG (40) CG (40)	AG (M 23; F 17) CG (M 14; F 26)	AG: 53.5 ± 11.0 CG: 54.7 ± 8.7	6

*AG *Accelerated Group, *CG *Conservative Group, *M *Males, *F *Females

^a^Data are reported as number of patients unless otherwise indicated

#### Clinical outcomes, range of motions and retear rate

As shown in Table 2, several clinical scores and ROMs have been measured in the included studies. Regarding clinical outcomes, the scoring systems usually used were the CMS measured in 9 out 16 studies [61, 63, 64, 68, 69, 72, 73, 77, 110], then SST score in 5 out 16 studies [63, 64, 68, 70, 110], and ASES score in 5 out 16 studies [63, 64, 68, 70, 77]. ROMs were reported in 14 out 16 studies [29, 61, 63, 64, 67, 68, 70, 72, 73, 77–80, 110].

**Table 2:** Clinical outcomes and range of motions at a maximum of follow-up

Authors (Year, level of evidence)	No of patients in the accelerated group (AG) and conservative group (CG)a	Clinical outcomes (Mean ± standard deviation)			Range of motion (Degrees ± standard deviation)					
		Constant-Murley score	Simple shoulder test	ASES score	Forward elevation	Internal rotation at side	External rotation at side	Internal rotation at 90° of abduction	External rotation at 90° of abduction	Abduction
Arndt et al. [61] (2012, II)	AG (49)	77.6 ± 12.4	-	-	172.4 ± 13.0	-	58.7 ± 12.9	-	-	-
	CG (43)	69.7 ± 18	-	-	163.3 ± 25.1	-	49.1 ± 18.0	-	-	-
Cuff et al. [70] (2012, I)	AG (33)	-	11.1	91.1	174	94	46	-	-	-
	CG (35)	-	11.1	92.8	173	91	45	-	-	-
De Roo et al. [110] (2015, I)	AG (79)	85.9 ± 15.7	9.4 ± 2.2	-	139.2 ± 16.8	-	46.2 ± 17.3	62.5 ± 16.8	64.2 ± 17.4	128 ± 20.2
	CG (51)	90.4 ± 18.4	9 ± 2.5	-	141.3 ± 17.7	-	46.3 ± 14.7	64.5 ± 21.3	63.53 ± 16.60	129.6 ± 18.7
Düzgün et al. [71] (2011, II)	AG (13)	-	-	-	-	-	-	-	-	-
	CG (16)	-	-	-	-	-	-	-	-	-
Düzgün et al. [29] (2014, II)	AG (19)	-	-	-	158.9 ± 1.8	88.2 ± 2.1	86.3 ± 2.4	-	-	173.6 ± 3.4
	CG (21)	-	-	-	153.7 ± 4	86.5 ± 1.9	84.1 ± 2.2	-	-	171 ± 3.1
Jenssen et al. [73] (2018, I)	AG (60)	86 ± 27	-	-	151 ± 15	61 ± 9	64 ± 12	-	-	146 ± 22
	CG (58)	90 ± 23	-	-	149 ± 15	62 ± 11	64 ± 11	-	-	146 ± 22
Keener et al. [63] (2014, I)	AG (61)	83.2 ± 11.5	10.8 ± 1.8	91.0 ± 15.3	164 ± 13.4	-	62.0 ± 16.4	-	-	-
	CG (53)	84.3 ± 10.8	10.6 ± 2.5	93.3 ± 10.6	163 ± 15.8	-	66.2 ± 14.0	-	-	-
Kim et al. [64] (2012, I)	AG (56)	69.8 ± 1.2	9 ± 0.9	73.3 ± 8.7	159.7 ± 4.8	-	78.5 ± 4	-	-	-
	CG (49)	69.9 ± 2.3	9 ± 0.8	82.9 ± 4.6	153.7 ± 3.9	-	81.3 ± 6.1	-	-	-
Klintberg et al. [72] (2009, II)	AG (7)	74.2 ± 12.9	-	-	146.3 ± 18.8	-	-	50 ± 8.7	80 ± 17.3	160 ± 20.2
	CG (7)	81 ± 8.6	-	-	150 ± 8.7	-	-	51.3 ± 10.1	61.3 ± 21.6	165 ± 11.5
Koh et al. [77] (2014, I)	AG (40)	85.6 ± 15.6	-	88.9 ± 16.2	143.7 ± 23.5	9.1 ± 2.9	33.1 ± 18.8	-	-	-
	CG (48)	88.7 ± 9.7	.-	92.1 ± 10.2	141.7 ± 25.2	9.4 ± 2.9	29.5 ± 21.2	-	-	-
Lee et al. [67] (2012, II)	AG (30)	-	-	-	155.3 ± 13.0	-	53.0 ± 11.6	65.7 ± 13.3	76.3 ± 12.1	167.8 ± 12.8
	CG (34)	-	-	-	153.0 ± 12.2	-	48.1 ± 13.9	54.9 ± 21.5	77.7 ± 11.6	161.8 ± 27.3
Mazzocca et al. [68] (2017, II)	AG (31)	82 ± 15	10.2 ± 2.6	90 ± 15	176 ± 11	-	61 ± 18	-	-	-
	CG (27)	75 ± 19	9.3 ± 3.6	84 ± 19	173 ± 17	-	58 ± 17	-	-	-
Raschhofer et al. [69] (2017, II)	AG (14)	78.8 ± 1.4	-	-	-	-	-	-	-	-
	CG (15)	67 ± 3.5	-	-	-	-	-	-	-	-
Sheps et al. [78] (2015, II)	AG (97)	-	-	-	149.9 ± 12.4	-	-	34.9 ± 9.7	81.5 ± 11.7	150.7 ± 20
	CG (92)	-	-	-	149.9 ± 17.8	-	-	35.7 ± 10.2	84.0 ± 11.9	155.5 ± 20.2
Sheps et al. [79] (2019, I)	AG (103)	-	-	-	155.5 ± 12.7	-	-	40.9 ± 12	76 ± 14.8	153.5 ± 14.6
	CG (103)	-	-	-	152.2 ± 18.5	-	-	38.7 ± 12.4	71.5 ± 17.8	152.2 ± 21.9
Tirefort et al. [80] (2019, I)	AG (40)	-	-	-	156.3 ± 18.8	-	-	-	39.3 ± 15.8	-
	CG (40)	-	-	-	153.3 ± 21.8	-	-	-	38.4 ± 20.4	-

*AG *Accelerated Group, *CG *Conservative Group, *M *Males, *F *Females, *ASES *American shoulder and elbow surgeons

^a^Data are reported as number of patients unless otherwise indicated

The outcome measures have been extracted by time-point follow-up from all the studies (Tables 3, 4, 5, 6, 7 and 8).

**Table 3:** Retear rate

Authors (Year, level of evidence)	No of patients in the accelerated group (AG) and conservative group (CG)^a^	No of retear (%)
Arndt et al. [61] (2012, II)	AG (49)	11 (23.3 %)
	CG (43)	7 (15.4 %)
Cuff et al. [70] (2012, I)	AG (33)	5 (15 %)
	CG (35)	3 (8 %)
De Roo et al. [110] (2015, I)	AG (79)	0 (0 %)
	CG (51)	2 (4 %)
Düzgün et al. [29] (2014, II)	AG (19)	0 (0 %)
	CG (21)	0 (0 %)
Keener et al. [63] (2014, I)	AG (61)	6 (10 %)
	CG (53)	3 (6 %)
Kim et al. [64] (2012, I)	AG (56)	7 (12 %)
	CG (49)	9 (18 %)
Koh et al. [77] (2014, I)	AG (40)	5 (12.5 %)
	CG (48)	4 (8.3 %)
Lee et al. [67] (2012, II)	AG (30)	7 (23 %)
	CG (34)	3 (8.8 %)
Mazzocca et al. [68] (2017, II)	AG (31)	11 (34 %)
	CG (27)	9 (31 %)
Sheps et al. [79] (2019, I)	AG (103)	5 (4.9 %)
	CG (103)	4 (3.9 %)

*AG *Accelerated Group, *CG *Conservative Group

^a^Data are reported as number unless otherwise indicated

**Table 4:** Clinical outcomes and ROMs at 3-month follow-up

Authors (Year, level of evidence)	No of patients in the accelerated group (AG) and conservative group (CG)^a^	Clinical outcomes (Mean ± standard deviation)			Range of motion (Degrees ± standard deviation)					
		Constant-Murley score	Simple shoulder test	ASES score	Forward elevation	Internal rotation at side	External rotation at side	Internal rotation in abduction	External rotation in abduction	Abduction
Arndt et al. [61] (2012, II)	**AG (49)**	-	-	-	142.1 ± 28.2	-	45.6 ± 14.9	-	-	-
	**CG (43)**	-	-	-	112.9 ± 37.6	-	27.5 ± 19.4	-	-	-
Düzgün et al. [29] (2014, II)	**AG (19)**	-	-	-	154.4 ± 2.2	80 ± 3.7	68.3 ± 5.3	-	-	166 ± 5.9
	**CG (21)**	-	-	-	141.4 ± 5.9	68.2 ± 3.3	58.6 ± 4.8	-	-	151.9 ± 5.3
Jenssen et al. [73] (2018, I)	**AG (60)**	41 ± 23	-	-	112 ± 31	47 ± 12	45 ± 19	-	-	101 ± 36
	**CG (58)**	38 ± 19	-	-	118 ± 27	48 ± 12	45 ± 14	-	-	102 ± 32
Keener et al. [63] (2014, I)	**AG (61)**	-	-	-	136 ± 23.6	-	47 ± 18.5	-	-	
	**CG (53)**	-	-	-	123 ± 30.6	-	40.1 ± 18.8	-	-	
Kim et al. [64] (2012, I)	**AG (56)**	63.2 ± 1.7	6.3 ± 0.6	65.2 ± 3	144.9 ± 2.8	-	71.2 ± 4.5	-	-	-
	**CG (49)**	63.3 ± 2.1	6.1 ± 0.7	64.7 ± 3.5	140 ± 3.9	-	66,3 ± 4.1	-	-	-
Lee et al. [67] (2012, II)	**AG (30)**	-	-	-	149.7 ± 12.7	-	44.2 ± 14.6	59 ± 17.9	70.5 ± 14	161.5 ± 22
	**CG (34)**	-	-	-	133.8 ± 27.4	-	34.1 ± 19.2	38.5 ± 24.1	54 ± 24.5	143.6 ± 35.7
Mazzocca et al. [68] (2017, II)	**AG (31)**	64 ± 12	7.4 ± 2.4	70 ± 13	168 ± 14	-	53 ± 13	-	-	-
	**CG (27)**	61 ± 15	5.6 ± 3.1	64 ± 18	167 ± 19	-	55 ± 19	-	-	-
Raschhofer et al. [69] (2017, II)	**AG (14)**	67.3 ± 6.1	-	-	-	-	-	-	-	-
	**CG (15)**	58.3 ± 2.6	-	-	-	-	-	-	-	-
Sheps et al. [78] (2015, II)	**AG (97)**	-	-	-	120 ± 25.4	-	-	23.7 ± 16	52.6 ± 29.10	112.1 ± 29.1
	**CG (92)**	-	-	-	118.5 ± 25.5	-	-	24.1 ± 13.8	53.7 ± 26.9	113 ± 26.20
Sheps et al. [79] (2019, I)	**AG (103)**	-	-	-	125.5 ± 28.5	-	-	30.2 ± 15.2	53 ± 27.6	119.1 ± 31.3
	**CG (103)**	-	-	-	121 ± 30.1	-	-	25.9 ± 16.3	44.2 ± 28.8	116 ± 37.6
Tirefort et al. [80] (2019, I)	**AG (40)**	-	-	-	125.8 ± 24.4	-	27.5 ± 18	-	-	-
	**CG (40)**	-	-	-	153.3 ± 21.8	-	34.1 ± 17.8	-	-	-

*AG *Accelerated Group, *CG *Conservative Group, *ASES *American shoulder and elbow surgeons

^a^Data are reported as number of patients unless otherwise indicated

**Table 5:** Clinical outcomes and ROMs at 6-month follow-up

Authors (Year, level of evidence)	No of patients in the accelerated group (AG) and conservative group (CG)^a^	Clinical outcomes (Mean ± standard deviation)			Range of motion (Degrees ± standard deviation)					
		Constant-Murley score	Simple shoulder test	ASES score	Forward elevation	Internal rotation at side	External rotation at side	Internal rotation in abduction	External rotation in abduction	Abduction
Arndt et al. [61] (2012, II)	AG (49)	-	-	-	158.4 ± 22.9	-	54.3 ± 12.5	-	-	-
	CG (43)	-	-	-	146.4 ± 30	-	44.3 ± 19.4	-	-	-
Cuff et al. [70] (2012, I)	AG (33)	-	-	-	172	79	44	-	-	-
	CG (35)	-	-	-	165	60	43	-	-	-
Düzgün et al. [71] (2011, II)	AG (13)	-	-	-	-	-	-	-	-	-
	CG (16)	-	-	-	-	-	-	-	-	-
Düzgün et al. [29] (2014, II)	AG (19)	-	-	-	158.9 ± 1.8	88.2 ± 2.1	86.6 ± 2.4	-	-	173.6 ± 3.4
	CG (21)	-	-	-	153.7 ± 4	86.5 ± 1.9	84.1 ± 2.2	-	-	171 ± 3.1
Jenssen et al. [73] (2018, I)	AG (60)	68 ± 28	-	-	138 ± 25	56 ± 10	57 ± 15	-	-	130 ± 33
	CG (58)	71 ± 25	-	-	141 ± 20	56 ± 14	59 ± 15	-	-	134 ± 30
Keener et al. [63] (2014, I)	AG (61)	74.4 ± 13.3	9.1 ± 2.7	81.1 ± 16.2	155 ± 18.1	-	61.6 ± 17.8	-	-	80 ± 14.10
	CG (53)	74.6 ± 11.3	9.3 ± 2.9	84.3 ± 15.1	154 ± 17.8	-	63.9 ± 15.1	-	-	81.3 ± 13
Kim et al. [64] (2012, I)	AG (56)	66.1 ± 1.7	7.8 ± 0.5	67.1 ± 3.1	150.6 ± 5.1	-	77.2 ± 3.1	-	-	-
	CG (49)	64.5 ± 2.1	6.7 ± 0.6	69.9 ± 3.3	147.1 ± 3.5	-	72.9 ± 4.9	-	-	-
Klintberg et al. [72] (2009, II)	AG (7)	63.5 ± 9.8	-	-	138.8 ± 15.9	-	-	49 ± 14.4	63.8 ± 21.6	157.75 ± 13
	CG (7)	64.8 ± 18.7	-	-	126.3 ± 30.3	-	-	41.3 ± 7.2	70 ± 17.3	142.5 ± 37.5
Lee et al. [67] (2012, II)	AG (30)	-	-	-	157.3 ± 11.4	-	50.3 ± 11.2	63.8 ± 14.3	74 ± 11	165.3 ± 13.9
	CG (34)	-	-	-	151.9 ± 18.2	-	41.6 ± 14.9	47.3 ± 22.7	67.8 ± 18.1	154.4 ± 30.1
Mazzocca et al. [68] (2017, II)	AG (31)	78 ± 11	10 ± 2.4	88 ± 16	173 ± 20	-	63 ± 16	-	-	-
	CG (27)	75 ± 15	8.7 ± 3.3	80 ± 19	173 ± 10	-	61 ± 17	-	-	-
Raschhofer et al. [69] (2017, II)	AG (14)	78.8 ± 1.4	-	-	-	-	-	-	-	-
	CG (15)	67 ± 3.5	-	-	-	-	-	-	-	-
Sheps et al. [78] (2015, II)	AG (97)	-	-	-	136.3 ± 18.1	-	-	32.2 ± 12.7	73.1 ± 17.3	134.6 ± 27.3
	CG (92)	-	-	-	135.9 ± 21.6	-	-	32.9 ± 12	74.2 ± 17.1	139.1 ± 25.8
Sheps et al. [79] (2019, I)	AG (103)	-	-	-	142.3 ± 22.6	-	-	35.9 ± 12.1	67.3 ± 21	139.4 ± 23.5
	CG (103)	-	-	-	141.3 ± 22.6	-	-	35.5 ± 12.2	62.9 ± 21.6	139.5 ± 27.60
Tirefort et al. [80] (2019, I)	AG (40)	-	-	-	156.3 ± 18.8	-	39.3 ± 15.8	-	-	-
	CG (40)	-	-	-	153.3 ± 21.8	-	38.4 ± 20.4	-	-	-
^a^Data are reported as number of patients unless otherwise indicated *AG *Accelerated Group, *CG *Conservative Group, *ASES *American shoulder and elbow surgeons										

*AG *Accelerated Group, *CG *Conservative Group, *ASES *American shoulder and elbow surgeons

^a^Data are reported as number of patients unless otherwise indicated

**Table 6:** Clinical outcomes and ROMs at 12-month follow-up

Authors (Year, level of evidence)	No of patients in the accelerated group (AG) and conservative group (CG)^a^	Clinical outcomes (Mean ± standard deviation)			Range of motion (Degrees ± standard deviation)					
		Constant-Murley score	Simple shoulder test	ASES score	Forward elevation	Internal rotation at side	External rotation at side	Internal rotation in abduction	External rotation in abduction	Abduction
Arndt et al. [61] (2012, II)	AG (49)	-	-	-	171.9 ± 13.6	-	58.1 ± 13.2	-	-	-
	CG (43)	-	-	-	161.9 ± 26.2	-	48.3 ± 18.2	-	-	-
Cuff et al. [70] (2012, I)	AG (33)	-	11.01	91.1	174	94	46	-	-	-
	CG (35)	-	11.01	92.8	173	91	45	-	-	-
Jenssen et al. [73] (2018, I)	AG (60)	86 ± 27	-	-	151 ± 15	61 ± 9	64 ± 12	-	-	146 ± 22
	CG (58)	90 ± 23	-	-	149 ± 15	62 ± 11	64 ± 11	-	-	146 ± 22
Keener et al. [63] (2014, I)	AG (61)	79.1 ± 10	10.3 ± 2.3	88.1 ± 15.8	161 ± 13.4	-	64.1 ± 15.2	-	-	84.7 ± 13.9
	CG (53)	79.9 ± 12.3	10 ± 3.1	89.1 ± 14.1	159 ± 22.8	-	67.3 ± 15.9	-	-	88.6 ± 11.9
Kim et al. [64] (2012, I)	AG (56)	69.8 ± 1.2	9 ± 0.9	73.3 ± 8.7	159.7 ± 4.8	-	78.5 ± 4	-	-	-
	CG (49)	69.9 ± 2.3	9 ± 0.8	82.9 ± 4.6	153.7 ± 3.9	-	81.3 ± 6.1	-	-	-
Klintberg et al. [72] (2009, II)	AG (7)	81 ± 8.6	-	-	146.3 ± 18.8	-	-	50 ± 8.7	80 ± 17.3	160 ± 20.2
	CG (7)	74.3 ± 13	-	-	150 ± 8.7	-	-	51.3 ± 10.1	61.3 ± 21.6	165 ± 11.5
Lee et al. [67] (2012, II)	AG (30)	-	-	-	155.3 ± 13	-	53 ± 11.6	65.7 ± 13.3	76.3 ± 12.1	167.8 ± 12.8
	CG (34)	-	-	-	153 ± 12.2	-	48.1 ± 13.9	54.9 ± 21.5	77.7 ± 11.6	161.8 ± 27.3
Mazzocca et al. [68] (2017, II)	AG (31)	82 ± 15	10.2 ± 2.6	90 ± 15	176 ± 11	-	61 ± 18	-	-	-
	CG (27)	75 ± 19	9.3 ± 3.6	84 ± 19	173 ± 17	-	58 ± 17	-	-	-
Sheps et al. [78] (2015, II)	AG (97)	-	-	-	144.8 ± 14.4	-	-	33.9 ± 9.9	77.9 ± 13.4	144.7 ± 22.6
	CG (92)	-	-	-	114.8 ± 19.2	-	-	34.8 ± 10.3	80.3 ± 13.9	149.9 ± 23.2
Sheps et al. [79] (2019, I)	AG (103)	-	-	-	150.9 ± 12.6	-	-	39 ± 12.4	75.3 ± 16.2	150 ± 15.3
	CG (103)	-	-	-	149 ± 19.9	-	-	40.1 ± 14.4	70.6 ± 18.8	148.4 ± 22.4

*AG *Accelerated Group, *CG *Conservative Group, *ASES *American shoulder and elbow surgeons

^a^Data are reported as number of patients unless otherwise indicated

**Table 7:** Clinical outcomes and ROMs at 24-month follow-up

Authors (Year, level of evidence)	No of patients in the accelerated group (AG) and conservative group (CG)^a^	Clinical outcomes (Mean ± standard deviation)			Range of motion (Degrees ± standard deviation)				
		Constant-Murley score	Simple shoulder test	ASES score	Forward elevation	External rotation at side	Internal rotation at 90° of abduction	External rotation at 90° of abduction	Abduction
Keener et al. [63] (2014, I)	AG (61)	83.2 ± 11.5	10.8 ± 1.8	91 ± 15.3	164 ± 13.4	62 ± 16.4	-	-	90 ± 10.3
	CG (53)	84.3 ± 10.8	10.6 ± 2.5	93.3 ± 10.6	163 ± 15.8	66.2 ± 14	-	-	87.7 ± 11.9
Klintberg et al. [72] (2009, II)	AG (7)	82.3 ± 6.1	-	-	-	-	-	-	-
	CG (7)	75.8 ± 11.8	-	-	-	-	-	-	-
Sheps et al. [78] (2015, II)	AG (97)	-	-	-	149.9 ± 12.4	-	34.9 ± 9.7	81.5 ± 11.7	150.7 ± 20
	CG (92)	-	-	-	149.9 ± 17.8	-	35.7 ± 10.2	84 ± 11.9	155.5 ± 20.2
Sheps et al. [79] (2019, I)	AG (103)	-	-	-	155.5 ± 12.7	-	40.9 ± 12	76 ± 14.8	153.5 ± 14.6
	CG (103)	-	-	-	152.2 ± 18.5	-	38.7 ± 12.4	71.5 ± 17.8	152.2 ± 21.9

*AG *Accelerated Group, *CG *Conservative Group, *ASES *American shoulder and elbow surgeons

^a^Data are reported as number of patients unless otherwise indicated

**Table 8:** VAS score at 3, 6, 12, and 24-month follow-up

Authors (Year, level of evidence)	No of patients in the accelerated group (AG) and conservative group (CG)^a^	VAS score (mean ± standard deviation)			
		3-month follow-up	6-month follow-up	12-month follow-up	24-month follow-up
Jenssen et al. [73] (2018, I)	AG (60)	-	-	8.6 ± 1.8	-
	CG (58)	-	-	8.7 ± 1.9	-
Keener et al. [63] (2014, I)	AG (61)	-	1.4 ± 1.6	1.1 ± 1.7	0.9 ± 1.7
	CG (53)	-	1.1 ± 1.4	0.9 ± 1.2	0.6 ± 1.1
Kim et al. [64] (2012, I)	AG (56)	-	3	2.8	-
	CG (49)	-	3.2	1.8	-
Klintberg et al. [72] (2009, II)	AG (7)	2.5 ± 0.5	1.2 ± 0.8	0	0.8 ± 0.9
	CG (7)	2.4 ± 1.7	0.3 ± 0.5	0.9 ± 1.1	0.3 ± 0.4
Koh et al. [77] (2014, I)	AG (40)	-	2.7 ± 1.5	-	1.3 ± 1.8
	CG (48)	-	2.7 ± 1.8	-	0.8 ± 1.0
Lee et al. [67] (2012, II)	AG (30)	-	-	0.9 ± 0.9	-
	CG (34)	-	-	0.8 ± 0.9	-
Mazzocca et al. [68] (2017, II)	AG (31)	1.6 ± 1.4	1.0 ± 1.7	0.7 ± 1.5	-
	CG (27)	2.7 ± 2.3	1.4 ± 2.3	0.9 ± 1.5	-
Raschhofer et al. [69] (2017, II)	AG (14)	1.9 ± 0.7	0.4 ± 0.4	-	-
	CG (15)	2 ± 0.8	0.1 ± 0.5	-	-
Sheps et al. [78] (2015, II)	AG (97)	1.4 ± 1.6	0.8 ± 1.3	0.5 ± 0.8	0.4 ± 0.9
	CG (92)	1.3 ± 1.6	0.9 ± 1.6	0.9 ± 1.7	0.5 ± 1.3
Sheps et al. [79] (2019, I)	AG (103)	1.8 ± 2.1	0.9 ± 1.3	0.6 ± 1.0	0.7 ± 1.3
	CG (103)	1.4 ± 1.6	0.8 ± 1.0	0.6 ± 1.0	0.6 ± 1.3
Tirefort et al. [80] (2019, I)	AG (40)	1.9 ± 1.9	0.8 ± 1.1	-	-
	CG (40)	2.6 ± 2.1	1.5 ± 1.6	-	-

*AG *Accelerated Group, *CG *Conservative Group, *ASES *American shoulder and elbow surgeons

^a^Data are reported as number of patients unless otherwise indicated

### Quality assessment results

The reliability between pairs was a substantial agreement with a kappa of 0.75 (*p*-value < 0.001; CI 95 %: 0.62–0.87). We judged ten out of 16 studies as having a low risk of bias for selection bias because they reported using an appropriate method to generate the allocation schedule [63, 68–70, 72, 73, 77–80]. Only one study was considered as high risk since a quasi-random sequence generation was used [62]. The allocation concealment was unclear in the remaining five studies [61, 64, 67, 71, 110].

Due to the lack of blinding of the patient and personnel, three studies were judged as high risk for performance bias [64, 68, 80]; whereas the blinding of participants and personnel was not described in six studies [61, 67, 70, 73, 78, 110]. Regarding the detection bias domain, we judged eleven studies as having a low risk of bias because of the blinding of outcome assessors [29, 63, 64, 68–70, 77–80, 110].

We judged three trials as having a high risk of bias for incomplete outcomes data since they reported more than 20 % loss to follow-up [67, 68] and unbalanced loss among the groups [78]. Two studies were judged as having a high risk of bias because outcomes were reported incompletely, so they cannot be entered in a meta-analysis [29, 70]. Other potential bias was not identified.

**Fig. 2:**
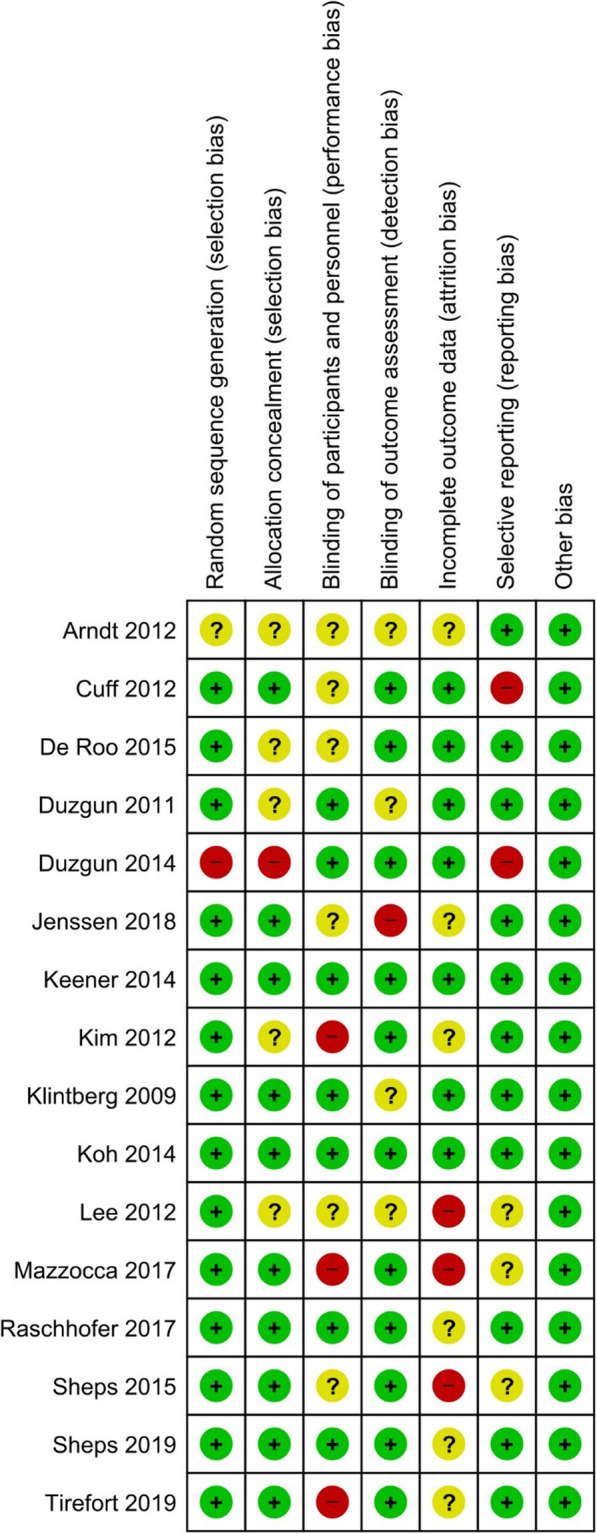
Risk of bias summary: review authors’ judgements about each risk of bias item for each included study

**Fig. 3:**
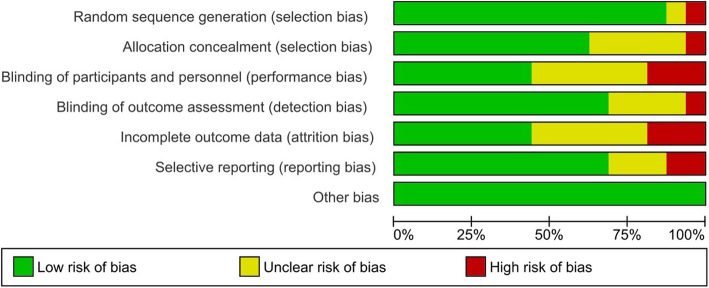
Risk of bias graph: review authors’ judgements about each risk of bias item presented as percentages across all included studies.

Please see the risk of bias summary presented in the figures (Figs. 2 and 3).

We provided an assessment of GRADE quality of evidence. The quality of the evidence of the included studies was found to be high.

### Meta-analysis

Each study evaluated the shoulder function through several outcomes (e.g., American Society of Shoulder and Elbow Surgeon, Simple Shoulder Test, Western Ontario Rotator Cuff Index, Disabilities of the Arm, Shoulder, and Hand Questionnaire, Range of Motion). Meta-analysis was performed to investigate potential differences between conservative and accelerated rehabilitation protocol after RC repair in retear rate, CMS and ROMs (forward elevation, external rotation, and abduction) at 3, 6, 12, and 24 months of follow-up (Table 9).

**Table 9:** Summary of meta-analysis data

Variables	Follow-up (months)	No. patients accelerated group	No. patients conservative group	Mean differences (95 % CI)	*p*-value
Forward elevation	3	546	520	4.71 (-0.82, 10.24)	0.10
	6	553	527	3.77 (2.65, 4.89)	< 0.001*
	12	494	466	6.43 (0.27, 12.60)	0.04*
	24	261	248	1.51 (-1.17, 4.19)	0.27
External rotation	3	346	325	5.41 (1.43, 9.39)	0.008*
	6	346	325	3.19 (2.20, 4.81)	< 0.001*
	12	287	264	1.31 (-2.55, 5.17)	0.51
	24	-	-	-	-
Abduction	3	309	308	6.61 (-1.55, 14.77)	0.11
	6	377	368	1.69 (-0.02, 3.41)	0.05*
	12	358	347	-1.48 (-4.22, 1.26)	0.29
	24	261	248	0.31 (-2.48, 3.11)	0.83
Constant-Murley Score	3	161	149	3.71 (-1.93, 9.36)	0.20
	6	229	209	2.79 (-2.91, 8.49)	0.34
	12	215	194	-0.07 (-0.77, 0.63)	0.84
	24	68	60	0.02 (-3.76, 3.80)	0.99

^*^indicates a statistically significant *p*-value

To determine the clinical reliability of findings arising from the quantitative analysis, thresholds for MCID were established a priori. In agreement with the literature, MCID for CMS was 6.3 points, whereas MCID for abduction, forward flexion, and external rotation were 7°, 12°, and 3°, respectively [111, 112].

#### Retear rate

**Fig. 4:**
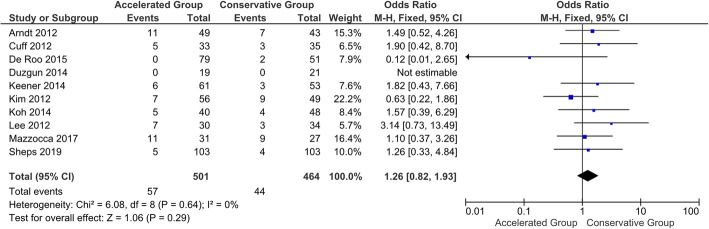
Forest plot of retear. No statistically significant differences were found in retear rate between patients who followed the accelerated rehabilitation protocol and those treated conservatively (*P* = 0.29; I^2^ = 0 %)*.*

Surgical failures were reported in 10 out 16 studies [29, 61, 63, 64, 67, 68, 70, 77, 79, 110]. Re-tears occurred in 44 of 464 (9.5 %) patients in the conservative group, whereas in 57 of 501 (11.4 %) patients in accelerated one (Table 3). The meta-analysis showed no statistically significant differences in terms of retear rate between patients who followed the accelerated rehabilitation protocol and those treated conservatively (*P* = 0.29) (Fig. 4) [113].

#### CMS and ROMs at 3-month follow-up

**Fig. 5:**
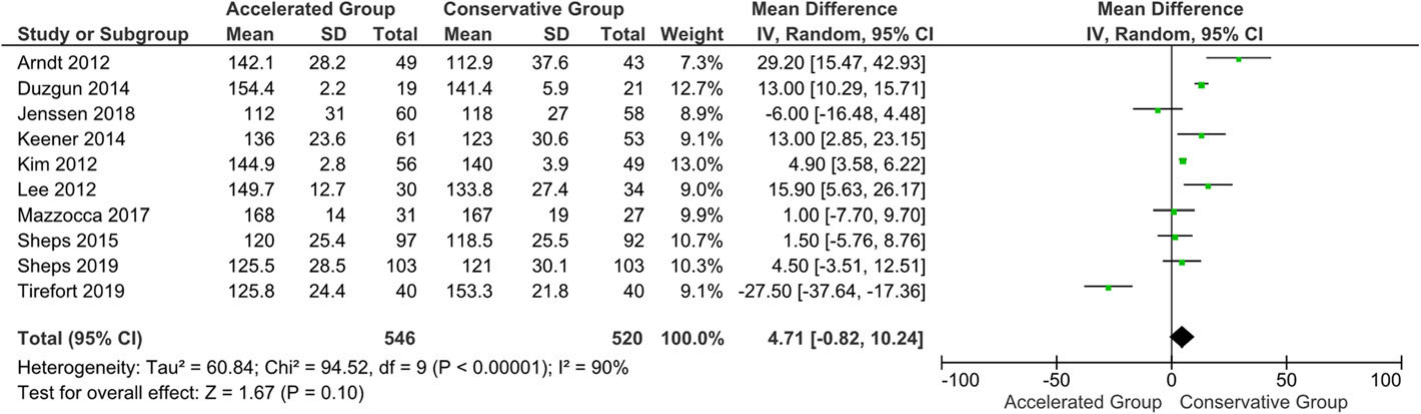
Forest plot of forward elevation at 3-month follow-up. No statistically significant differences were found in forward elevation between patients who followed the accelerated rehabilitation protocol and those treated conservatively (*P* = 0.1; I^2^ = 90 %)

**Fig. 6:**
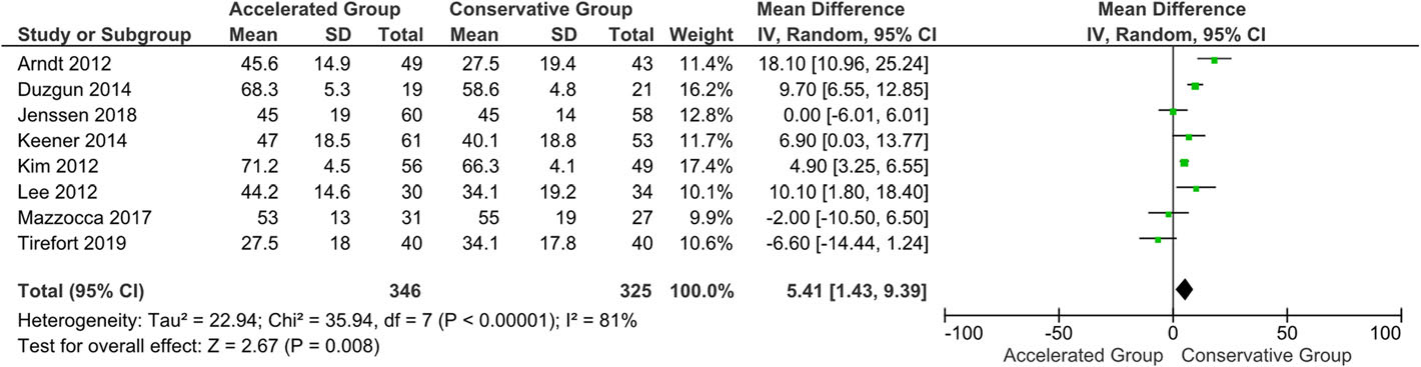
Forest plot of external rotation at 3-month follow-up. The accelerated group provide better results in external rotation than conservative group (*P* < 0.05; I^2^ = 81 %)

**Fig. 7:**
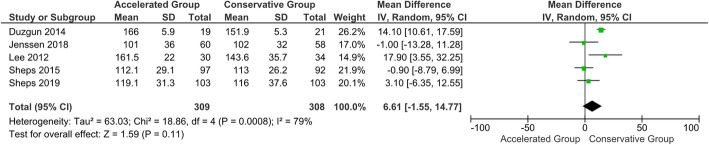
Forest plot of abduction at 3-month follow-up. No statistically significant differences were found in abduction between patients who followed the accelerated rehabilitation protocol and those treated conservatively (*P* = 0.11; I^2^ = 79 %)

No statistically significant difference in CMS was shown at the 3-month follow-up (*P* = 0.20). Furthermore, the quantitative analysis showed no difference between the groups in terms of abduction and forward flexion. On the other hand, the accelerated group provided statistically and clinically better results in terms of external rotation than the conservative group (*P* < 0.05) (Figs. 5, 6 and 7). The 3-month follow-up data extracted from all the included studies are shown in Table 4 [113].

#### CMS and ROMs at 6-month follow-up

**Fig. 8:**
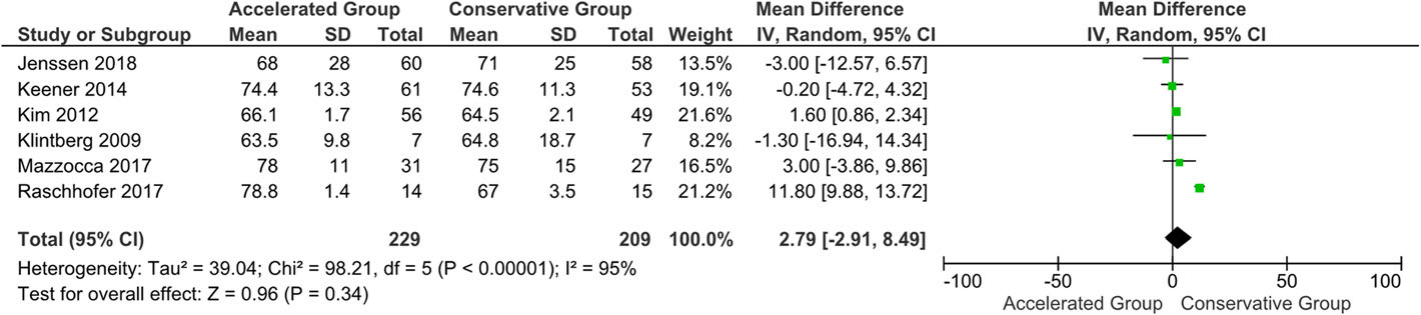
Forest plot of CMS at 6-month follow-up. No statistically significant differences were found in Constant-Murley score between patients who followed the accelerated rehabilitation protocol and those treated conservatively (*P* = 0.34; I^2^ = 95 %)

**Fig. 9:**
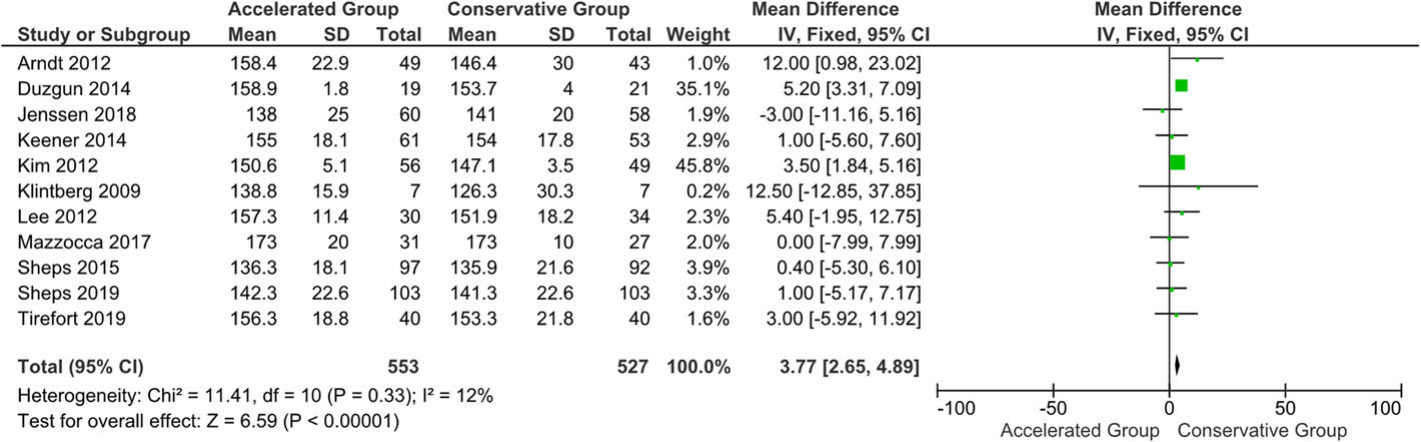
Forest plot of forward elevation at 6-month follow-up. The accelerated group provide better results in forward elevation than conservative group (*P* < 0.05; I^2^ = 12 %)

**Fig. 10:**
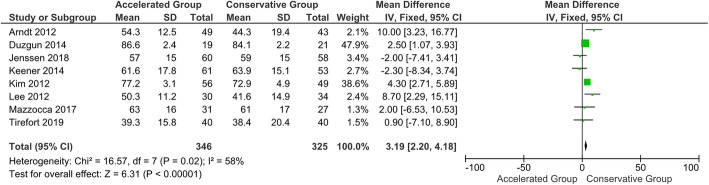
Forest plot of external rotation at 6-month follow-up. The accelerated group provide better results in external rotation than conservative group (*P* < 0.05; I^2^ = 58 %)

No statistically significant difference in CMS was shown at the 6-month follow-up (*P* = 0.34). Significant statistical differences were found in favour of the accelerated group in terms of forward elevation, external rotation and abduction at 6-month follow-up (*P* < 0.05) (Figs. 8, 9 and 10). However, based on the a priori established MCID threshold, clinical reliability was only found for external rotation movement. The 6-month follow-up data extracted from all the included studies are shown in Table 5 [113].

#### CMS and ROMs at 12-month follow-up

**Fig. 11:**
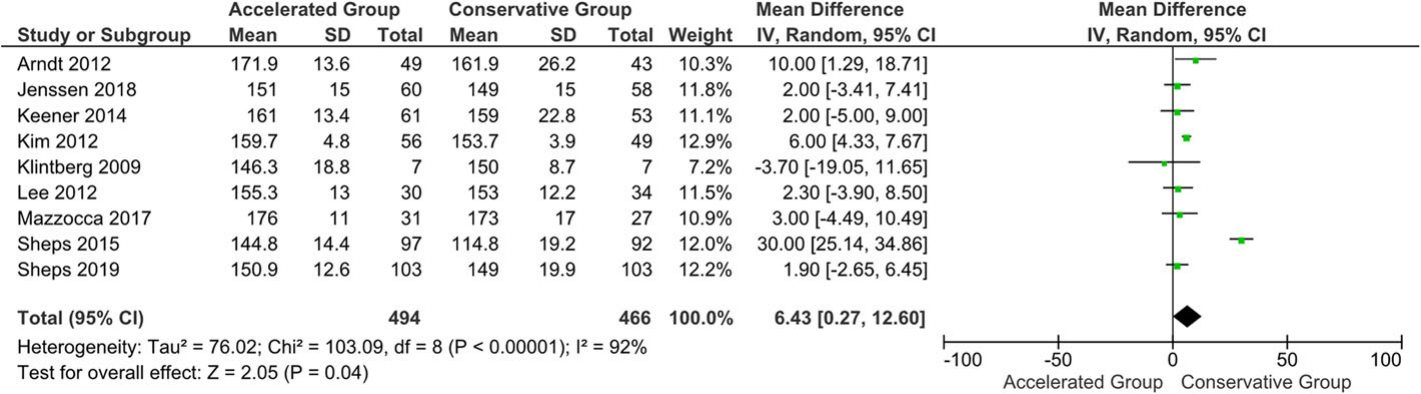
Forest plot of forward elevation at 12-month follow-up. The accelerated group provide better results in forward elevation than conservative group (*P* < 0.05; I^2^ = 92 %)

No statistically significant difference in CMS was shown at the 12-month follow-up (*P* = 0.84). One year after the intervention, the superiority of the accelerated group was found only in terms of forward elevation (*P* < 0.05) (Fig. 11). However, based on the a priori established MCID threshold, clinical reliability can not be sustained. All 12-month follow-up data are described in Table 6 [113].

#### CMS and ROMs at 24-month of follow-up

**Fig. 12:**
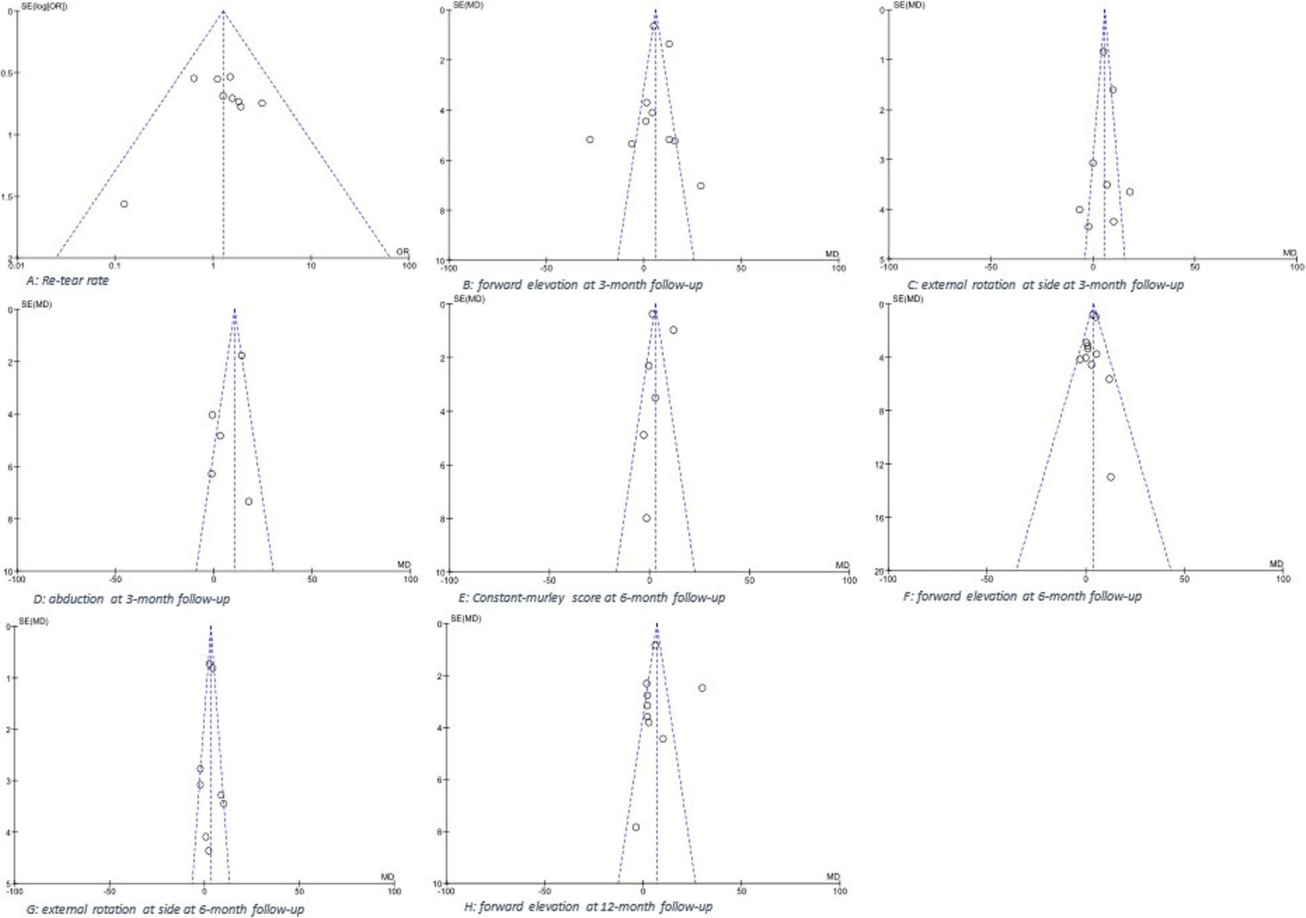
Funnel plots of comparisons: conservative versus accelerated rehabilitation protocols

Only 4 out of 16 studies conducted a 24-month follow-up (Table 7). The meta-analysis showed no significant differences between the two group in terms of CMS or ROMs. The funnel plots of all comparisons are shown in Fig. 12.

## Discussion

Pros and cons of accelerated and conservative rehabilitation protocols after arthroscopic RC repair have been reported [4, 53, 92–98]. Based on the available evidence, the American Academy of Orthopaedic Surgeons (AAOS) could not draw definitive conclusions for the best rehabilitation protocol [114]. The purpose of the present systematic review was to identify a potential superiority of either form of rehabilitation in clinical outcomes and ROMs at 3, 6, 12, and 24-month follow-up. The meta-analysis highlighted clinically and statistically significant differences in favour of accelerated rehabilitation in terms of external rotation at three months of follow-up. The conservative group demonstrated worse results in terms of abduction, forward elevation and external rotation at six months follow-up than the accelerated group. However, regarding clinical interpretability, thresholds for MCID were achieved only for external rotation. The quantitative analysis showed a mean difference for forward elevation and abduction of 3.77 and 1,61, respectively. Despite their statistical significance, these results may lack clinical relevance because they do not exceed MCID. Besides, forward elevation at 12-month follow-up was statistically but not clinically superior in the accelerated group than in the conservative one. Only four studies recorded two-year follow-up revealing comparable efficacy among accelerated and conservative rehabilitation protocols [63, 72, 78, 79].

During the first postoperative weeks, the tendon healing process occurs through the first inflammatory phase (first week), followed by the second stage of cell proliferation (second and third week) and, finally, the maturation and restoration to original condition period (up to 6 months) [115, 116]. Shoulder immobilization has always been considered a critical step in the treatment protocol to balance the preservation of repair integrity and restoration of function in patients undergoing RC repair [26, 74, 117]. However, histopathological changes responsible for joint stiffness arising from prolonged immobilization have been demonstrated [115, 118, 119]. To avoid the negative effect of immobilization and to support a quick reintegration into daily activities, an accelerated protocol enabling movements before six weeks can be recommended [42, 64, 71]. However, the first clinical results revealed that patients following an accelerated rehabilitation protocol experienced an increased number of retear events [120–122]. Among the ten included studies that reported retear events, only Arndt et al., Cuff et al., and Lee et al. demonstrated a slight and not statistically significant improvement in the tendon healing process with immobilization [61, 67, 70]. However, based on both our results and those of the previous meta-analyses, there are no statistically significant differences in the retear rate among the accelerated and conservative groups [53, 65, 66, 93].

This systematic review included 14 studies providing different ROMs results between conservative and accelerated rehabilitation groups after RC repair. The majority of available studies have shown comparable results among the groups [29, 63, 64, 70, 73, 77, 79, 110]. Interestingly, when data has stratified for time-points follow-up, Arndt et al., Lee et al., and Tirefort et al. reported improvements in shoulder function at 6-month follow-up in patients who performed accelerated rehabilitation [61, 67, 80]. Moreover, according to the findings of this meta-analysis, the accelerated group’s superiority in terms of external rotation was demonstrated both at three and 6-month follow-up. Previous studies have shown that the tendon healing process requires the first six postoperative months and that several variables which may occur during this period can influence the outcome. [75, 76, 115, 116, 123]. In the short-term, our results in terms of ROMs were demonstrated by a previous meta-analysis [96].

Interestingly, when the comparison was adjusted for the tear size (i.e. large lesion), Chang et al. observed a statistically significant trend towards an increased number of retear events in the accelerated group [96]. Therefore, as tear size, several other factors may influence the early postoperative rehabilitation outcomes [124]. Indeed, minor injuries are related to all ROMs’ progress, large lesions with all ROMs except flexion [65, 124]. However, the potential role of the tear size is still debate. As regards small and medium lesions (< 5 cm), the arm immobilization for six weeks following arthroscopic RC treatment seems to improve tendon healing [125]. However, prolonged immobilization did not provide significant shoulder function advantages [67, 77, 126, 127]. Even though the accelerated rehabilitation protocol seems to improve ROMs, a decrease in tendon healing has been reported in tears sized 3–5 cm [128]. Therefore, the impact of tear size in terms of clinical outcomes requires further investigation.

The present meta-analysis showed a reduction of statistically significant results in the long term. A better forward flexion at 12-month follow-up in favour of accelerated rehabilitation protocol was detected. However, the clinical reliability of this finding is weak. Among the ten studies that made the comparison one year after surgery, only Arndt et al. reported slightly worse results for the conservative group in terms of external rotation [61]. In contrast, the remaining studies found comparable effects on final shoulder function. Comparable findings were assessed at 24 months of follow-up between the groups suggesting that the two rehabilitation protocols could be equally safe and effective. However, this result may be influenced by the lack of studies with long-term follow-up. To date, only four high-level studies have analysed the effectiveness of the two forms of rehabilitation protocol two years after the surgery without showing statistically significant results [63, 72, 78, 79]. Therefore, further long-term studies are essential to define the superiority of one of the two forms of rehabilitation protocol.

Although rigorous methods were used for this systematic review, and only level I-II studies were selected to increase the strength of the results, some limitations should be outlined. Whereas the conservative protocol applied was the same in all studies, the accelerated group performed protocols with different exercises and sessions per week (Table 10). In several studies, the first postoperative movements were performed with pendulum exercises or passive mobilization with rope, pulley, or cane. A comparison of these exercises was not possible. Besides, active mobilization was started at different time points in different studies. In the immediate postoperative period, only two studies allowed active shoulder motion [64, 68]. In the other studies, active ROMs were allowed at three week [29, 73], at four week [72, 80], at five week [110], and at six week postoperatively [61, 63, 67, 70], whereas in three studies the beginning of active ROMs was unspecified or unclear [69, 77, 79]. Shoulder muscle strengthening was allowed after 4–6 weeks postoperatively in four studies [62, 67, 69, 72], after 8–9 weeks in two studies [64, 110], after three months in three studies [63, 70, 80], after four months in two studies [61, 77], while in the remaining three it was not specified or was unclear [68, 73, 79]. Given this wide heterogeneity, it was not possible to perform a sub-analysis between protocols. Therefore, we are unable to quantify whether these differences may have influenced our results. To decrease the sample population’s heterogeneity, only level I-II studies evaluating the two forms of rehabilitation after arthroscopic RC repair were examined. However, due to the lack of information on the RC tear characteristics (e.g. tear size) in many studies, we could not conduct a subgroup analysis. Furthermore, the RC tear’s chronicity or the level of muscle atrophy and fatty infiltration was not specified in most of the included articles. Moreover, those articles that provided these data different, and not comparable, classification systems were used.

**Table 10:** Comparison of accelerated rehabilitation protocols

Authors	Accelerated rehabilitation protocol
**Arndt et al.** [61]	Shoulder passive ROM and pendulum exercise 1 d postoperatively (3–5 times/wk); active shoulder rehabilitation 6 wk postoperatively; shoulder muscle strengthening 4 mo postoperatively
**Cuff et al.** [70]	Shoulder passive ROM and pendulum exercise 2 d postoperatively (3 times/ wk and 3 times/d, respectively); active ROM of elbow, wrist, and hand immediately after surgery; aa shoulder exercise 6 wk postoperatively (3 times/wk); rotator cuff strengthening 12 wk postoperatively
**De Roo et al.** [110]	Shoulder passive ROM and pendulum exercises 1 d postoperatively (3–5 times/wk). Specific capsular glenohumeral exercises and progressively active ROM 5 wk postoperatively. Rotator cuff strengthening 8 wk postoperatively
**Düzgün et al.** [29, 71]	Shoulder passive ROM 1 wk postoperatively (1 time/d at clinic and 2 times/d at home); active ROM of elbow, hand, and neck 1 wk postoperatively; active shoulder rehabilitation 3 wk postoperatively; rotator cuff strengthening 4 wk postoperatively
**Jenssen et al.** [73]	Shoulder passive ROM and pendulum exercises 1 d postoperatively; shoulder active ROM 3 wk postoperatively
**Keener et al.** [63]	Pendulum exercise and active ROM of elbow, wrist, and hand immediately after surgery; shoulder passive ROM 1 wk postoperatively; shoulder active ROM 6 wk postoperatively; shoulder muscle strengthening 3 mo postoperatively; full activity 4 mo postoperatively
**Kim et al.** [64]	Shoulder passive ROM 2 d postoperatively; shrugging shoulder exercise and active ROM of elbow, forearm, wrist, and hand immediately after surgery; aa shoulder exercise after weaning of immobilizer; shoulder muscle strengthening 9 wk postoperatively; sport activities 6 mo postoperatively
**Klintberg et al.** [72]	low-level active ROM 2 d postoperatively; active assisted ROM 4 weeks, aquatic training, loading at 6 weeks
**Koh et al.** [77]	4 weeks of immobilization without passive ROM; shoulder passive ROM with rope, pulley, and cane 4 wk postoperatively; shoulder muscle strengthening 11 wk postoperatively
**Lee et al.** [67]	Shoulder passive ROM exercise up to tolerable angle 1 d postoperatively (2 times/d by physical therapist and 3 times/d by patient, respectively); shoulder active ROM 6 wk postoperatively; shoulder muscle strengthening 6 wk postoperatively; recreational activities with heavy demands 6 mo postoperatively
**Mazzocca et al.** [68]	Shoulder active ROM with a cane 2–3 d postoperative
**Raschhofer et al.** [69]	Shoulder isometric activation (i.e. the dynamic relocation test) 2–6 wk postoperatively; shoulder muscle strengthening 6 wk postoperatively
**Sheps et al.** [78, 79]	Shoulder passive ROM and pain-free activities only, with the exception of resisted activities, at discharge
**Tirefort et al.** [80]	Shoulder passive ROM during the first 4 postoperative weeks. Active ROM 4 wk postoperatively; demanding activities and light sports were authorized after 2 months, and a strengthening program was permitted only after 3 postoperative months.

*ROM *Range of motion, *d *day, *wk *week, *mo *month

## Conclusions

No statistically significant differences in the retear rate among the accelerated and conservative group have been demonstrated. On the other hand, statistically and clinically significant differences were found in terms of external rotation at 3 and 6 months of follow-up in favour of the accelerated group. However, no differences between the two groups were detected at 24 months follow-up. Further long-term studies are warranted to define the superiority between the accelerated and conservative rehabilitation protocol.

## Data Availability

The datasets used and/or analysed during the current study available from the corresponding author on reasonable request.

## Abbreviations

RC: Rotator cuff
ROM: Range of motion
MeSH: Medical Subject Headings
CMS: Constant-Murley score
SST: Simple Shoulder Test
ASES: American Shoulder and elbow surgeons
VAS: Visual analog scale
AAOS: American Academy of Orthopaedic Surgeons
MCID: Minimal clinically important difference
